# Trends, patterns and relationship of antimicrobial use and resistance in bacterial isolates tested between 2015–2020 in a national referral hospital of Zambia

**DOI:** 10.1371/journal.pone.0302053

**Published:** 2024-04-16

**Authors:** Misheck Shawa, Atmika Paudel, Herman Chambaro, Harvey Kamboyi, Ruth Nakazwe, Luke Alutuli, Tuvshinzaya Zorigt, Taona Sinyawa, Mulemba Samutela, Joseph Chizimu, Manyando Simbotwe, Kyoko Hayashida, Naganori Nao, Masahiro Kajihara, Yoshikazu Furuta, Yasuhiko Suzuki, Hirofumi Sawa, Bernard Hang’ombe, Hideaki Higashi

**Affiliations:** 1 Hokudai Center for Zoonosis Control in Zambia, Hokkaido University, Lusaka, Zambia; 2 Division of Infection and Immunity, International Institute for Zoonosis Control, Hokkaido University, Sapporo, Japan; 3 GenEndeavor LLC, Hayward, CA, United States of America; 4 Central Veterinary Research Institute, Ministry of Fisheries and Livestock, Lusaka, Zambia; 5 Department of Pathology and Microbiology, University Teaching Hospital, Lusaka, Zambia; 6 Department of Pharmacy, University Teaching Hospital, Lusaka, Zambia; 7 Department of Biomedical Sciences, School of Health Sciences, University of Zambia, Lusaka, Zambia; 8 Zambia National Public Health Institute, Ministry of Health, Lusaka, Zambia; 9 Department of Disease Control, School of Veterinary Medicine, University of Zambia, Lusaka, Zambia; 10 Division of Collaboration and Education, International Institute for Zoonosis Control, Hokkaido University, Kita-ku, Sapporo, Japan; 11 Division of International Research Promotion, International Institute for Zoonosis Control, Hokkaido University, Kita-ku, Sapporo, Japan; 12 One Health Research Center, Hokkaido University, Kita-ku, Sapporo, Japan; 13 Toyota Central R&D Labs., Inc., Nagakute, Japan; 14 Division of Bioresources, International Institute for Zoonosis Control, Hokkaido University, Kita-ku, Sapporo, Japan; 15 International Collaboration Unit, International Institute for Zoonosis Control, Hokkaido University, Kita-ku, Sapporo, Japan; 16 Institute for Vaccine Research and Development (HU-IVReD), Hokkaido University, Kita-ku, Sapporo, Japan; 17 Division of Molecular Pathobiology, International Institute for Zoonosis Control, Hokkaido University, Kita-ku, Sapporo, Japan; 18 Africa Centre of Excellence for Infectious Diseases of Humans and Animals, School of Veterinary Medicine, University of Zambia, Lusaka, Zambia; 19 Department of Para-clinical Studies, School of Veterinary Medicine, University of Zambia, Lusaka, Zambia; University of Tripoli, LIBYA

## Abstract

Increased antimicrobial resistance (AMR) among bacteria underscores the need to strengthen AMR surveillance and promote data-based prescribing. To evaluate trends and associations between antimicrobial usage (AMU) and AMR, we explored a dataset of 34,672 bacterial isolates collected between 2015 and 2020 from clinical samples at the University Teaching Hospital (UTH) in Lusaka, Zambia. The most frequently isolated species were *Escherichia coli* (4,986/34,672; 14.4%), *Staphylococcus aureus* (3,941/34,672; 11.4%), and *Klebsiella pneumoniae* (3,796/34,672; 10.9%). Of the 16 drugs (eight classes) tested, only amikacin and imipenem showed good (> 50%) antimicrobial activity against both *E*. *coli* and *K*. *pneumoniae*, while nitrofurantoin was effective only in *E*. *coli*. Furthermore, 38.8% (1,934/4,980) of *E*. *coli* and 52.4% (2,079/3,791) of *K*. *pneumoniae* isolates displayed multidrug resistance (MDR) patterns on antimicrobial susceptibility tests. Among *S*. *aureus* isolates, 44.6% (973/2,181) were classified as methicillin-resistant (MRSA). Notably, all the MRSA exhibited MDR patterns. The annual hospital AMR rates varied over time, while there was a weak positive relationship (r = 0.38, 95% CI = 0.11–0.60) between the monthly use of third-generation cephalosporins (3GCs) and 3GC resistance among *Enterobacterales*. Overall, the results revealed high AMR rates that fluctuated over time, with a weak positive relationship between 3GC use and resistance. To our knowledge, this is the first report to evaluate the association between AMU and AMR in Zambia. Our results highlight the need to strengthen antimicrobial stewardship programs and optimize AMU in hospital settings.

## Introduction

Therapeutic challenges related to antimicrobial-resistant bacteria raise considerable concern worldwide. Many experts regard antimicrobial resistance (AMR) as a clear and present danger, projected to kill 10 million people annually by 2050 [1]. To help mitigate the global AMR crisis, the 68^th^ World Health Assembly in May 2015 adopted five strategic objectives (SOs) from the Global Action Plan [2]. The second SO involves research and surveillance, which enables the development of focused intervention policies to guide antimicrobial prescribing decisions and limit AMR spread. AMR surveillance encompasses phenotypic and genotypic methods, each with specific strengths and limitations. For instance, whole-genome sequencing (WGS) can track AMR spread, but this approach is limited to known AMR genes and mutations. Furthermore, the presence of an AMR gene may not necessarily translate to phenotypic resistance if the gene is truncated or harbors frameshift mutations [3].

In contrast, phenotypic tests cannot evaluate AMR spread patterns but demonstrate a microbe’s response to an antibiotic, with direct clinical implications relevant to patient treatment. Furthermore, phenotypic tests might not detect inducible resistance when AMR genes are expressed only after prolonged exposure to antibiotics [4]. Therefore, an ideal surveillance system should utilize both molecular and phenotypic methods for better effectiveness. Yet, due to cost constraints and other limitations, only one approach is often adopted. Professional agencies such as the World Health Organization (WHO) recommend traditional antimicrobial susceptibility tests (AST) for AMR surveillance [5]. Similarly, the European Antimicrobial Resistance Surveillance Network [6] and the US National Antimicrobial Resistance Monitoring System [7] employ AST in their routine programs. Consequently, most developed countries have well-established AST-based monitoring programs aligned with these international bodies.

Although none of the 47 WHO members from Africa had launched comprehensive surveillance systems by 2017 [8], the political will among African countries is slowly growing. This is evidenced by the development of AMR national action plans by several member states, including Ghana, Liberia, Burkina Faso, Ethiopia, Kenya, Malawi, and South Africa [9]. Zambia launched its One Health Surveillance Platform for AMR in 2020 [10], supported by over ten hospitals that perform laboratory-based AST during routine operations. Local researchers have utilized the data generated by these hospitals to develop institutional antibiograms for clinicians in the country. For example, Roth et al. (2021) recently published treatment guidelines for infectious disease specialists and other practitioners based on susceptibility data recorded at the University Teaching Hospital (UTH) from 2015 to 2017 [11].

While AMR is a broad phenomenon encompassing various microbes, *Enterobacterales* resistant to third-generation cephalosporins (3GCs) are among the most dreaded because of their associated high mortality in humans [12,13]. However, factors driving the emergence of 3GC resistance are still poorly understood. This study aimed to evaluate the resistance trends and patterns among bacteria tested from 2015 to 2020. Furthermore, the relationship between 3GC usage and resistance among *Enterobacterales* was also explored.

## Methods

### Study site

The UTH is the largest hospital in Zambia, with a bed capacity of 1655. Being the highest-level referral hospital, the UTH provides services to all ten provinces, yielding results generalizable to the country. We obtained data on AMR in bacterial species and AMU between 2015 and 2020 at the UTH. The study period was chosen based on data available at the time of collection in September 2021. The data was previously collected using the UTH’s robust Laboratory Information System that uses Data Intensive Systems and Applications, making the data uniform despite being entered by different personnel over time.

### Analysis of AST data

To identify AST patterns at the UTH, raw data was collected for microorganisms isolated at the Microbiology Laboratory from 2015 to 2020 (S1 Table). The UTH ensured patient anonymity by unlinking personal data from patient identifiers prior to availing data for analysis. The data included microbial species and their AST results previously obtained by the VITEK 2 compact (bioMe’rieux, France), using VITEK® 2 AST-GN86 and AST-GP67 cards for Gram-negative and Gram-positive bacteria, respectively. Quality control of these VITEK cards was performed using the standard laboratory strain of *Escherichia coli* 25922. A cascade testing of antibiotics was used, and the breakpoints, which were based on the Clinical and Laboratory Standards Institute guidelines, were constant over the years. However, when VITEK cards were unavailable, ASTs were performed manually by disk diffusion, though the frequency of such occurrences was not determined. Furthermore, the antibiotics used for disk diffusion were based on the availability of disks.

**Fig 1:**
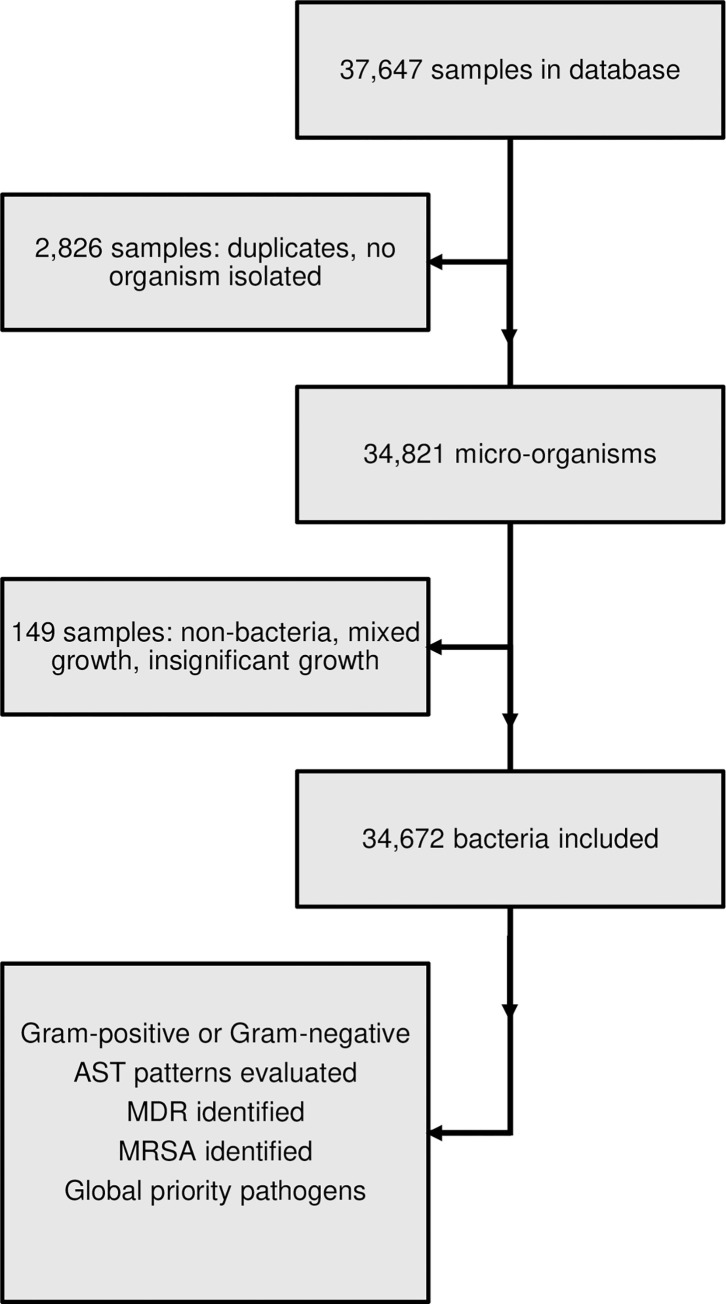
Study flowchart. The figure shows the collection and analysis of data on bacterial strains isolated at the UTH Microbiology Laboratory. Duplicates and samples with insignificant or no growth, no bacteria, and mixed growth were excluded from the dataset. The information was used to identify Gram-positive and Gram-negative bacteria, MDR, MRSA, and WHO global priority pathogens.

Normal flora (e.g., *E*. *coli* in stool samples) that are not routinely reported were not part of the data. Also, non-bacteria (e.g., *Cryptococcus*, *Candida*, *Prototheca*, etc.) were excluded from the data pool, along with samples having mixed or insignificant growth. Furthermore, duplicated data was trimmed to one entry, and samples with no organisms isolated were removed from the dataset (Fig 1). The bacteria were then classified as either Gram-positive or Gram-negative, and the frequency of each species was computed. Data manipulation was achieved with the R packages dplyr v1.0.7 [14] and reshape2 v1.4.4 [15], and AMR patterns for selected species were visualized using ggplot2 v3.3.5 [16]. Categorical variables were assessed by Chi-square if all the values of the generated 2 x 2 Table were ≥ 5, while the Fisher’s exact test was used whenever this condition was not fulfilled. Furthermore, odds ratios were determined with the epi.2by2 command of the epiR package. Finally, numeric variables were analyzed using Pearson’s product moment correlation in R.

To identify multidrug resistance (MDR), drugs were grouped by antimicrobial class, and multidrug-resistant isolates were defined as those resistant to one or more agents from at least three categories [17]. Furthermore, methicillin-resistant *Staphylococcus aureus* (MRSA) was defined as *S*. *aureus* isolates with reported resistance to cefoxitin (FOX) [18]. Finally, global priority pathogens listed by the WHO [19] were characterized by resistance to a representative antibiotic for each drug class. Based on testing frequency, imipenem (IPM) represented carbapenems, while ciprofloxacin (CIP) and cefotaxime (CTX) represented fluoroquinolones and 3GCs, respectively.

### Association of AMU with AMR

To quantify the usage of antimicrobials, we extracted hospital pharmacy data on the antibiotics dispensed monthly to the medical wards (E-block, Internal Medicine) in 2016, 2017, 2018, and 2020. Notably, there was no available data for 2015 and 2019 due to technical and human resource challenges. The medical wards were conveniently selected due to the availability of patient admission records. Using the Anatomical Therapeutic Chemical classification system [20], AMU was expressed as the Defined Daily Dose (DDD) per 100-patient days. Due to the cascade testing of antibiotics, AST data included antibiotics from the same class. Therefore, to avoid double-counting, AMR to 3GCs was defined as the percentage of isolates resistant to cefotaxime (CTX); CTX was chosen as a representative 3GC based on the testing frequency and clinical usage.

### Ethical issues

Ethical clearance was obtained from the Excellence in Research Ethics and Science (ERES; Reference number 2015-Feb-018) Converge. Furthermore, permission to use data was obtained from the UTH management, Ministry of Health under the Government of the Republic of Zambia. This study is part of the Government policy on monitoring AMR trends in Zambia. Patient anonymity was ensured by unlinking personal data from patient identifiers.

## Results

### Species distribution varied among clinical sources

**Fig 2:**
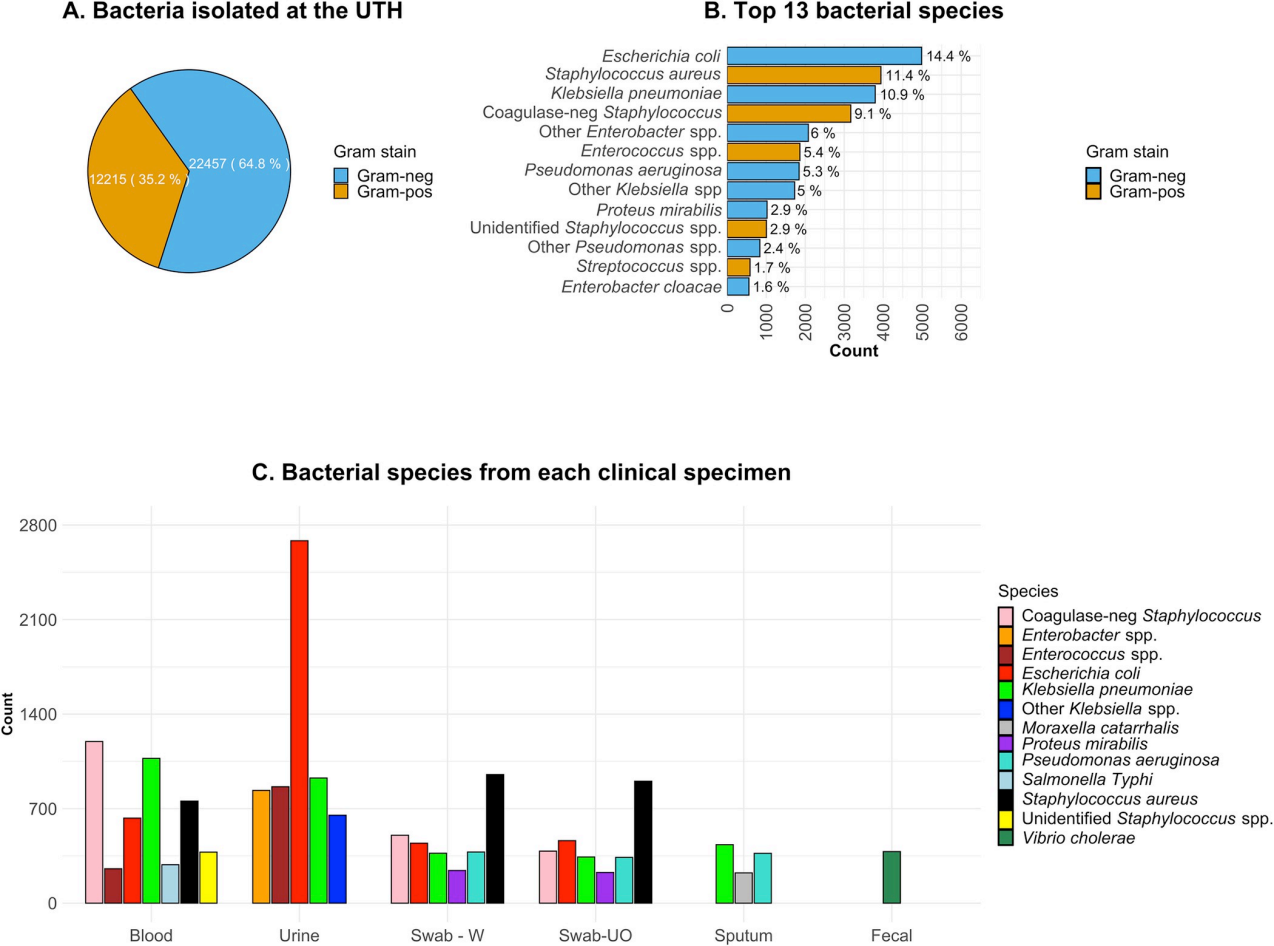
Characteristics of bacteria in the study. A. Classification of the isolated bacteria based on Gram-staining results. B. The top 13 organisms in the dataset. A cutoff prevalence of > 1.5% was used for the selection. C. Distribution of species across clinical samples. Normal flora organisms (e.g., *E*. *coli* in stool) were not included. Swab-W; wound swab. Swab-UO; swab with unknown origin.

From the review of UTH microbiology data, we found that 34,672 bacterial isolates were collected between 2015 and 2020, with Gram-negative species (22,457/34,672; 64.8%) being more abundant (Fig 2A, S2 Table). Overall, *E*. *coli* was the most predominant (4,986/34,672; 14.4%), followed by *S*. *aureus* (3,941/34,672; 11.4%) and *Klebsiella pneumoniae* (3,796/34,672; 10.9%) (Fig 2B). Of the 34,672 isolates analyzed, 28,336 (81.7%) had details on the clinical source, the majority being urine (8,082/28,336; 28.5%), blood (6,728/28,336; 23.7%), wound swabs (4,328/28,336; 15.3%), and swabs of unknown origin (4,222/28,336; 14.9%) (Fig 2C).

Bacteremia cases were mainly caused by coagulase-negative *Staphylococci* (1,197/6,728; 17.8%), *K*. *pneumoniae* (1,072/6,728; 15.9%), *S*. *aureus* (756/6,728; 11.2%), and *E*. *coli* (630/6,728; 9.4%) (S3 Table). While a number of studies report *E*. *coli* in over 50% of bacteriuria cases [21,22], in this study, *E*. *coli* accounted for 33.2% of the cases (2,684/8,082). Meanwhile, *S*. *aureus* dominated wounds (952/4,328; 22.0%) and swabs of unknown origin (903/4,222; 21.4%). Furthermore, *S*. *aureus* had the highest frequency among Gram-positive species, with most of them isolated from wound swabs (952/3,117; 30.5%) and swabs of unknown origin (903/3,117; 29.0%). In contrast, coagulase-negative *Staphylococci* were more abundant in blood samples (1,197/2,524; 47.4%), with much lower frequencies in wound swabs (503/2,524; 19.9%) and swabs of unknown origin (385/2,524; 15.3%) (S3 Table). Meanwhile, most *Enterococcus* species were isolated from urine samples (862/1,530; 56.3%), with a much lower frequency in blood (255/1,530; 16.7%). Of the 862 *Enterococcus* isolates from urine, *E*. *faecalis* alone accounted for 29.7% (256/862). Finally, several bacterial species of public health concern were isolated, including 384 *Vibrio cholerae* strains from stool and 285 *Salmonella enterica* subsp. *enterica* serovar Typhi isolates from blood samples (Fig 2C).

### AMR rates were high among the isolates

To determine the effectiveness of antibiotics, the AST results of the most isolated organisms were reviewed. The analysis showed that most drugs were less than 50% effective against the most frequently detected Gram-negative species, i.e., *E*. *coli* and *K*. *pneumoniae* (Table 1). The least effective drug was ampicillin (AMP), with only 6.8% (205/3,018) *E*. *coli* isolates being susceptible to this drug. β-lactamase inhibitors restore susceptibility to β-lactam antibiotics by irreversibly inhibiting β-lactamases [23]. Accordingly, the average proportion of *E*. *coli* (201/707, 28.4%) susceptible to AMP/sulbactam was higher than that for AMP alone. Moreover, this proportion was comparable to what we observed for co-amoxiclav (amoxicillin/clavulanic acid or AMX/CLA); 27.9% (150/538) for *E*. *coli*. The susceptibility pattern to AMX alone was not available in our data.

**Table 1:** Antimicrobial susceptibility pattern of *E*. *coli* and *K*. *pneumoniae*.

	*E*. *coli*			*K*. *pneumoniae*		
Drug	Tested	S	S (%)^†^	Tested	S	S (%)^†^
Ampicillin	3018	205	6.8	N/A	N/A	N/A
Ampicillin/sulbactam	707	201	28.4	738	146	19.8
Co-amoxiclav	538	150	27.9	648	126	19.4
Cefotaxime	847	344	40.6	847	179	21.1
Ceftazidime	1067	324	30.4	1451	224	15.4
Ceftriaxone	871	295	33.9	1143	166	14.5
Co-trimoxazole	1741	235	13.5	1543	154	10.0
Gentamicin	1915	954	49.8	2396	831	34.7
Nalidixic acid	1609	381	23.7	671	166	24.7
Norfloxacin	2107	876	41.6	875	350	40.0
Ciprofloxacin	1807	701	38.8	2247	819	36.4
**Amikacin**	**514**	**361**	**70.2**	**514**	**340**	**66.1**
**Imipenem**	**810**	**772**	**95.3**	**1369**	**1299**	**94.9**
Tobramycin	445	217	48.8	527	133	25.2
Tetracycline	517	101	19.5	505	165	32.7
**Nitrofurantoin** ^‡^	**2632**	**1750**	**66.5**	**1539**	**553**	**35.9**

^†^An antibiotic was considered effective if > 50% of the tested isolates were susceptible.

^‡^Nitrofurantoin was effective against *E*. *coli* but not against *K*. *pneumoniae*, N/A; Not Applicable.

S; susceptible.

Equally, 3GCs showed poor activity against Gram-negative species, with only a few isolates susceptible to CTX, ceftazidime (CAZ), and ceftriaxone (CRO) (Table 1). In addition, most non-β-lactam drugs also displayed low activity levels, as observed for co-trimoxazole (sulfamethoxazole/trimethoprim or SXT), tetracycline, gentamicin (GEN), nalidixic acid, norfloxacin, and CIP. On the other hand, two drugs showed good (> 50%) activity against *E*. *coli* and *K*. *pneumoniae*. Specifically, amikacin (AMK) exhibited effectiveness in over two-thirds of the isolates, while IPM was active in about 95%. Furthermore, nitrofurantoin (NIT) was active against 66.5% (1,750/2,632) of *E*. *coli* isolates, but its effectiveness against *K*. *pneumoniae* was only 35.9% (553/1,539) (Table 1).

Notably, the number of drugs effective against *S*. *aureus* was higher than those against *E*. *coli* and *K*. *pneumoniae* (Table 2). Generally, β-lactam antibiotics were ineffective, displaying antimicrobial activity in less than 50% of the tested *S*. *aureus* isolates. For instance, penicillin (PEN) and AMP were only effective in 11.6% (321/2,760) and 20.1% (38/189) of the isolates, respectively, while CTX and CRO had less than 50% effectiveness. However, unlike the observations made in *E*. *coli* and *K*. *pneumoniae*, a combination of AMX and CLA was effective in over 50% of the *S*. *aureus* isolates. Additionally, most non-β-lactam antibiotics were active against these isolates, with the highest effectiveness observed for linezolid (LZD) (424/451; 94.0%), vancomycin (VAN) (284/303; 93.7%), and NIT (323/346; 93.3%) (Table 2).

**Table 2:** Antimicrobial susceptibility pattern of *S*. *aureus*.

Drug	Tested	S	S (%)^†^
Ampicillin	189	38	20.1
Penicillin	2760	321	11.6
Co-amoxiclav	376	240	63.8
Cefotaxime	742	312	42.0
Chloramphenicol	1332	1054	79.1
Ceftriaxone	383	181	47.3
Co-trimoxazole	1477	363	24.6
Gentamicin	2408	1491	61.9
Clindamycin	1314	978	74.4
Levofloxacin	238	153	64.3
Ciprofloxacin	2507	1413	56.4
Amikacin	242	175	72.3
**Linezolid**	**451**	**424**	**94.0**
**Vancomycin**	**303**	**284**	**93.7**
Tetracycline	620	333	53.7
**Nitrofurantoin**	**346**	**323**	**93.3**
Erythromycin	2946	1427	48.4
Moxifloxacin	190	129	67.9

^†^An antibiotic was considered effective if > 50% of the tested isolates were susceptible.

S; susceptible.

### High MDR rates were detected among the isolates

To assess the level of MDR, the proportion of isolates resistant to at least one antibiotic from three or more drug classes was evaluated. We found that 38.8% (1,934/4,980) of *E*. *coli* and 52.4% (2,079/3,791) of *K*. *pneumoniae* isolates exhibited MDR phenotypes (Table 3). In addition, the MDR frequencies were significantly higher in blood samples compared to urine for both *E*. *coli* (Odds Ratio (OR) = 2.12, 95% CI = 1.76–2.56) and *K*. *pneumoniae* (OR = 8.18, 95% CI = 6.66–10.05) (S4 Table).

**Table 3:** MDR prevalence among *E*. *coli*, *K*. *pneumoniae*, and *S*. *aureus*.

**Species**	**Total screened**	**MDR phenotype (%)**		
		**Yes**		**No**
*E*. *coli*	4980	1934 (38.8)		3046 (61.2)
*K*. *pneumoniae*	3971	2079 (52.4)		1892 (47.6)
*S*. *aureus* (overall)	3420	1460 (42.7)		1960 (57.3)
*S*. *aureus* (MRSA)	973	973 (100)		0 (0)
*S*. *aureus* (MSSA)	1178	175 (14.9)		1003 (85.1)
*S*. *aureus* (FOX-Int)	30	10 (33.3)		20 (66.7)
		MRSA antibiotic susceptibility		
Drug	Total tested	S (%)	I (%)	R (%)
Ciprofloxacin	749	258 (34.4)	70 (9.3)	421(56.2)
Gentamicin	716	304 (42.5)	59 (8.2)	353 (49.3)
Clindamycin	364	208 (57.1)	53 (14.6)	103 (28.3)
Chloramphenicol	298	205 (68.8)	24 (8.1)	69 (33.7)
**Linezolid**	**183**	**166 (90.7)**	**1 (0.5)**	**16 (8.7)**
**Nitrofurantoin**	**75**	**69 (92.0)**	**3 (4.0)**	**3 (4.0)**
**Vancomycin**	**76**	**71 (93.4)**	**0 (0.0)**	**5 (6.6)**
Oxacillin	112	4 (3.6)	0 (0.0)	108 (96.4)

S; susceptible. I; intermediate. R; resistant.

For *S*. *aureus*, the overall MDR prevalence was 42.7% (1,460/3,420), with higher odds of occurrence in blood than wound swabs (OR = 1.25, 95% CI = 1.02–1.53) (S4 Table). To identify MRSA, the susceptibility of the isolates to FOX was assessed. Of the 2,181 isolates with FOX AST results, 973 (44.6%) were classified as MRSA, while 1,178 (54.0%) were sensitive to FOX and categorized as methicillin-susceptible *S*. *aureus* (MSSA) (Table 3). Further analysis showed that while all 973 (100%) MRSA exhibited MDR, only 14.9% (175/1,178) MSSA displayed MDR. Despite the high MDR prevalence among MRSA, some drugs showed high activity against these isolates. Specifically, VAN was effective in 93.4% (71/76) MRSA isolates, with NIT and LZD showing 92.0% (69/75) and 90.7% (166/183) effectiveness, respectively (Table 3).

### AMR rates varied over time

**Fig 3:**
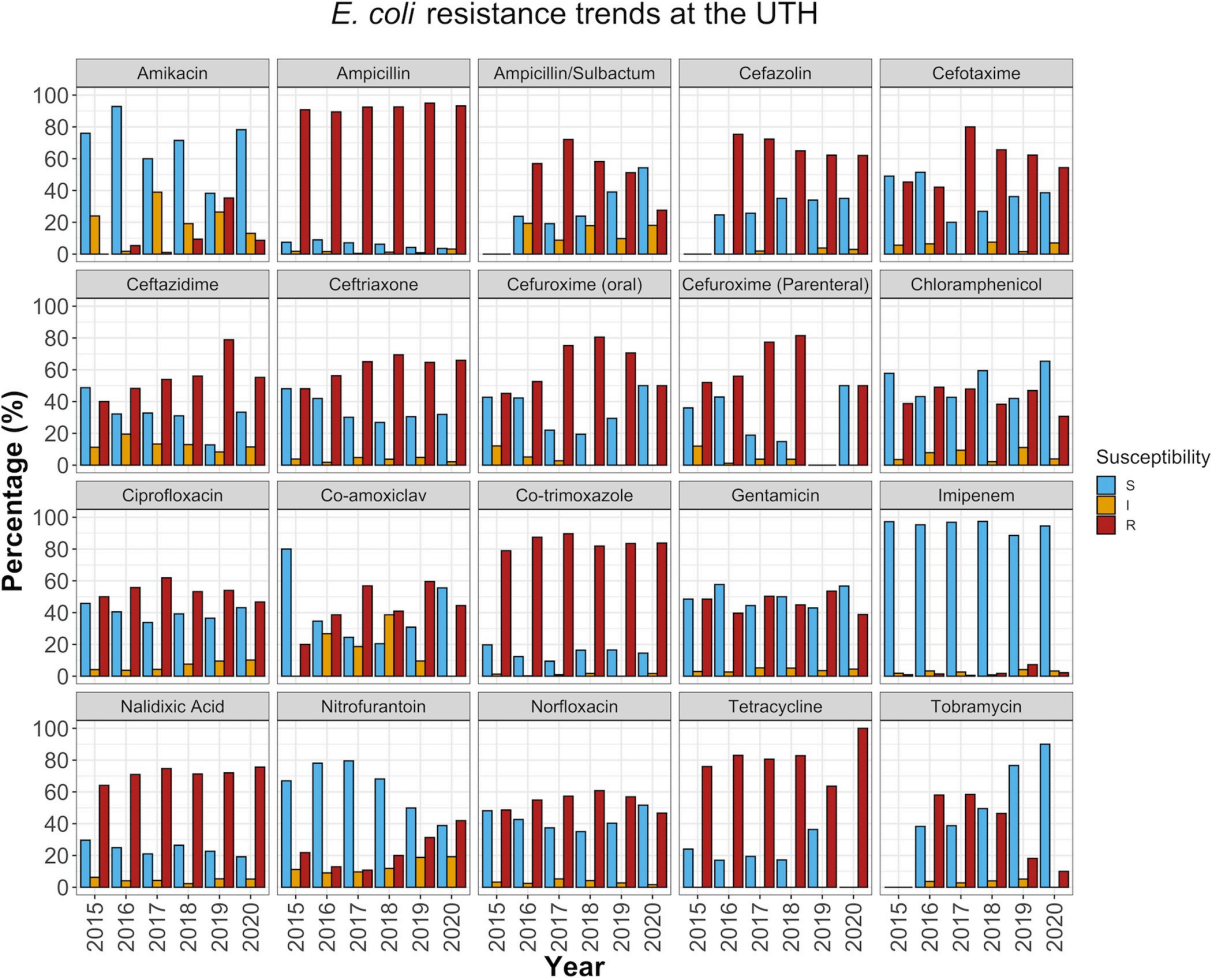
AST pattern for *E*. *coli*. The data was segmented into one-year intervals from 2015 to 2020. The plot represents the percentage of *E*. *coli* isolates susceptible, resistant, or exhibiting intermediate resistance to various antimicrobial drugs. The blue bars show the overall effectiveness of the drugs in a particular year. S (blue bars); susceptible strains. I (orange bars); strains with intermediate resistance; R (red bars); resistant strains.

**Fig 4:**
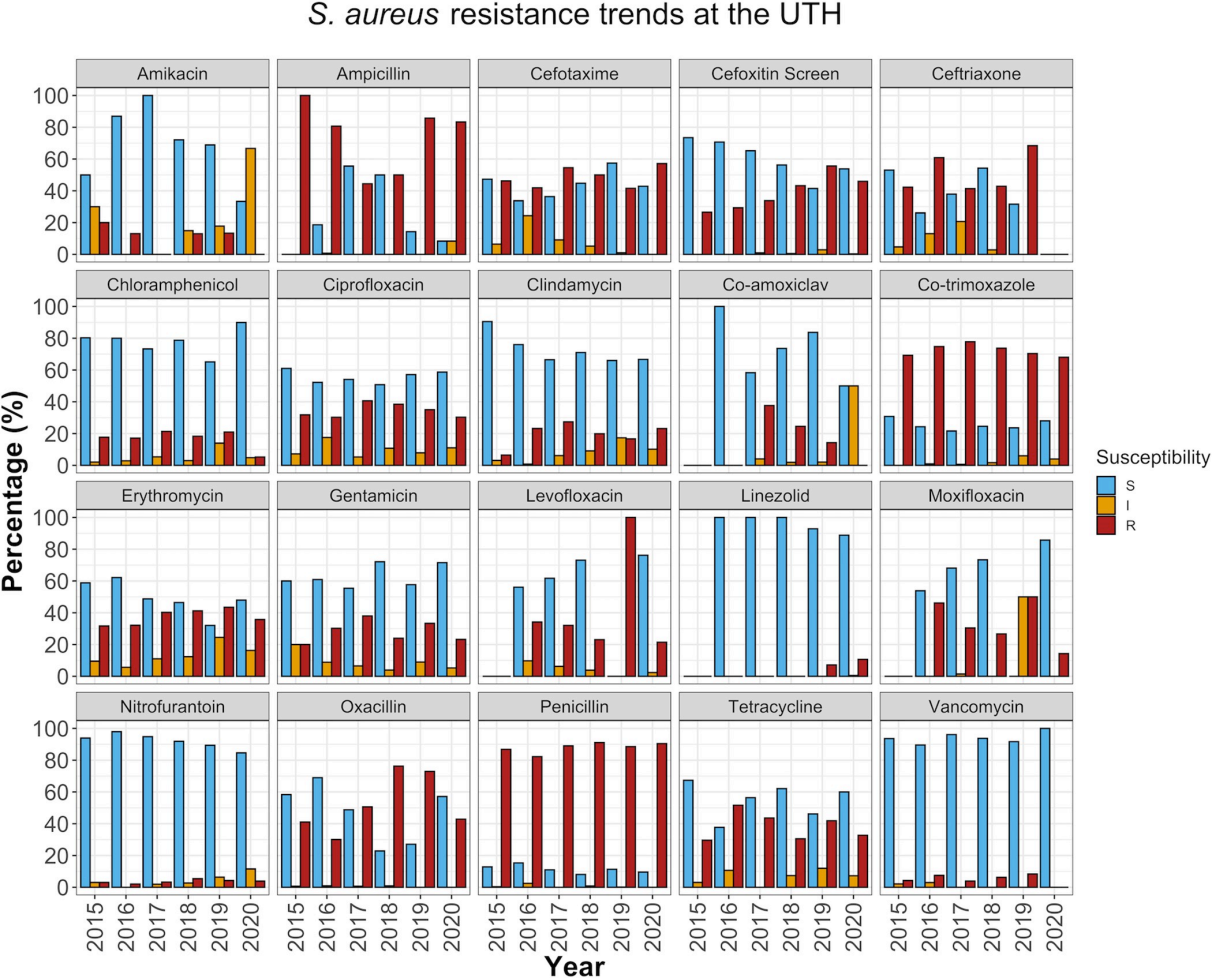
AST pattern for *S*. *aureus*. The figure represents the percentage of *S*. *aureus* isolates susceptible, resistant, or exhibiting intermediate resistance to various antimicrobial drugs. The blue bars show the overall effectiveness of the drugs in a particular year. S (blue bars); susceptible strains. I (orange bars); strains with intermediate resistance; R (red bars); resistant strains.

To determine the annual trends in AMR patterns over the study period, we stratified the antibiotic susceptibilities of the isolates by year. We found that some drugs that displayed good (> 50%) activity overall had lost effectiveness over time. For example, NIT, which showed an overall efficacy of 66.5% (1750/2632) in *E*. *coli* (Table 1), was more effective in the first half of the study period, with a peak effectiveness of 79.5% (435/547) in 2017. However, NIT effectiveness reduced to 38.8% (101/260) over the next three years (2018 to 2020) (*P* < 0.001) (Fig 3, S5 Table). A downward trend was also observed in *S*. *aureus*, where NIT effectiveness reduced from a peak of 98.0% (48/49) in 2016 to 84.6% (22/26) by 2020 (*P* = 0.046) (Fig 4, S5 Table).

**Fig 5:**
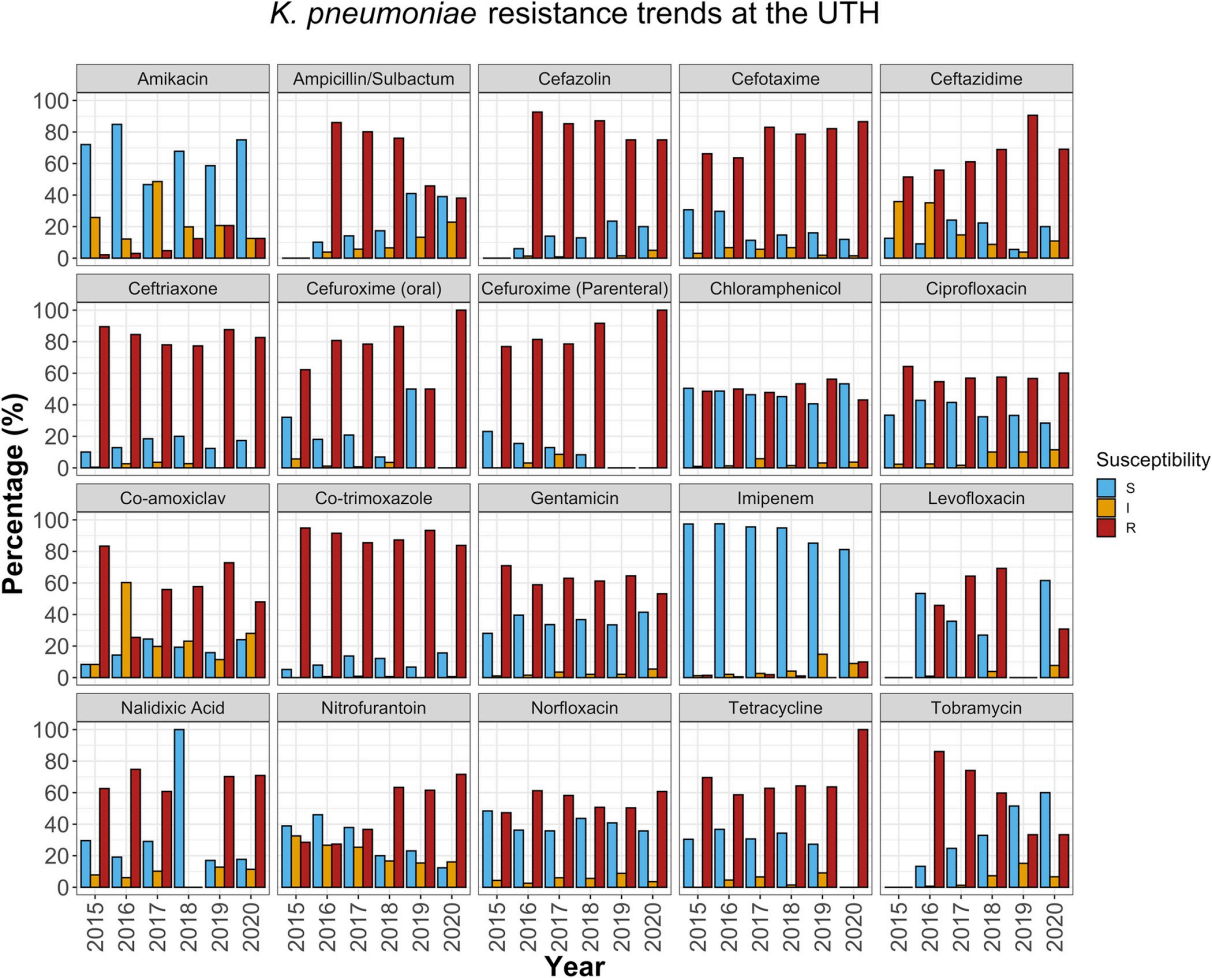
AST pattern for *K*. *pneumoniae*. The plot shows the percentage of *K*. *pneumoniae* isolates susceptible, resistant, or exhibiting intermediate resistance to various antimicrobial drugs. The blue bars represent the overall effectiveness of the drugs in a particular year. S (blue bars); susceptible strains. I (orange bars); strains with intermediate resistance; R (red bars); resistant strains.

On the other hand, GEN activity rarely exceeded 50% and 40% in *E*. *coli* (Fig 3) and *K*. *pneumoniae* (Fig 5), respectively. Furthermore, while GEN and kanamycin (KAN) were dispensed during the study period (S6 Table), susceptibility to tobramycin (TOB) increased between 2017 and 2020, from around 40% to 90% in *E*. *coli* (Fig 3) and 23% to 60% in *K*. *pneumoniae* (Fig 5). This is consistent with classic research on aminoglycoside resistance, which suggests limited cross-resistance among class members [24], with TOB showing potency in isolates resistant to GEN or KAN [25].

**Fig 6:**
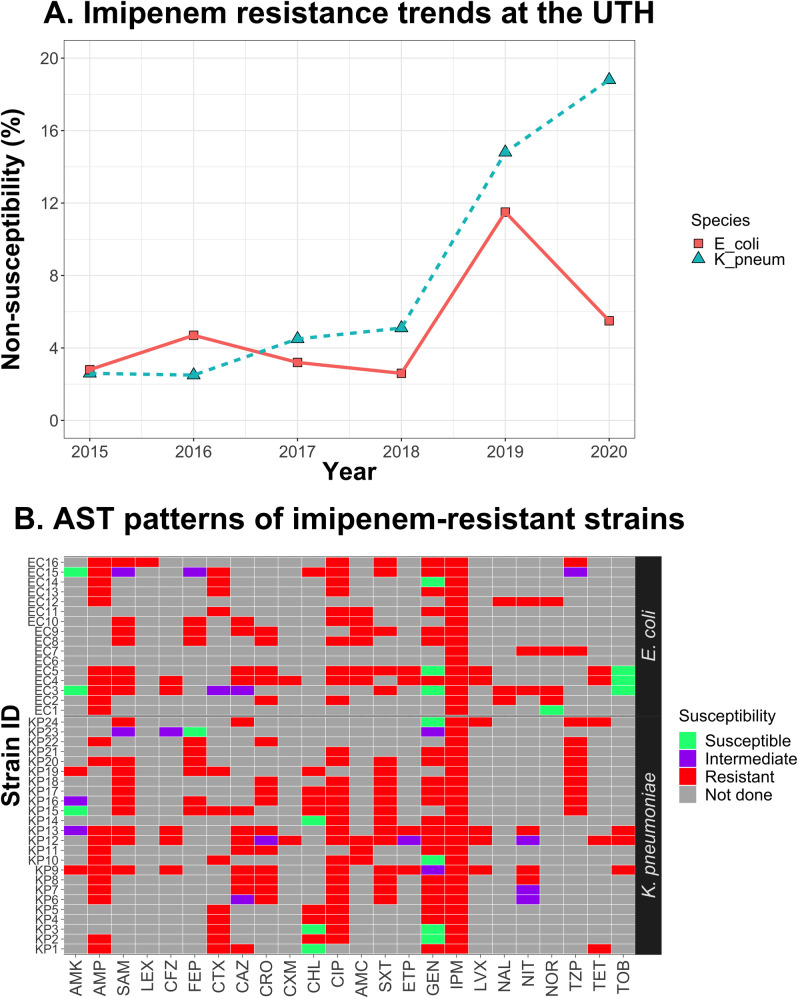
IPM non-susceptibility in *E*. *coli* and *K*. *pneumoniae*. A. Assessment of IPM efficacy in *E*. *coli* and *K*. *pneumoniae* from 2015 to 2020. Red squares and turquoise triangles represent the percentage of imipenem non-susceptible (i.e., resistant or intermediate) strains of *E*. *coli* and *K*. *pneumoniae*, respectively, per year. B. Susceptibility patterns of IPM-resistant isolates against other antimicrobial drugs.

Furthermore, we observed an increase in MRSA prevalence, typically detected based on FOX resistance [18]. Specifically, FOX effectiveness, at 73.5% (36/49) in 2015, decreased progressively, reaching 41.5% (344/829) in 2019 (*P* < 0.001) and 53.8% (226/420) in 2020 (*P* = 0.013) (S5 Table). Finally, we also observed a drop in IPM effectiveness against *E*. *coli* (*P* = 0.012) and *K*. *pneumoniae* (*P* = 0.048) in 2019. Although susceptibility was restored in *E*. *coli* in 2020 (*P* = 0.306), IPM effectiveness against *K*. *pneumoniae* reduced further (*P* = 0.006) (Fig 6A, S5 Table). Overall, IPM non-susceptibility was observed in 108 isolates, of which 40 (37%) were resistant, and 68 (63%) exhibited intermediate susceptibility. Previous studies have recommended that patients infected with strains displaying intermediate susceptibility be treated with a higher dose of the same drug [26], but resistant isolates necessitate using a different antibiotic. Therefore, we explored the 40 IPM-resistant isolates for possible alternative agents. We noted that only 35% (14/40) were susceptible to at least one agent, although no defined pattern existed. Nevertheless, IPM-resistant *E*. *coli* isolates tested against AMK (n = 2) and TOB (n = 3) were susceptible to these drugs; however, these sample sizes were too low to allow any meaningful interpretation of the data (Fig 6B).

### Correlation between 3GC usage and resistance among *Enterobacterales*

**Fig 7:**
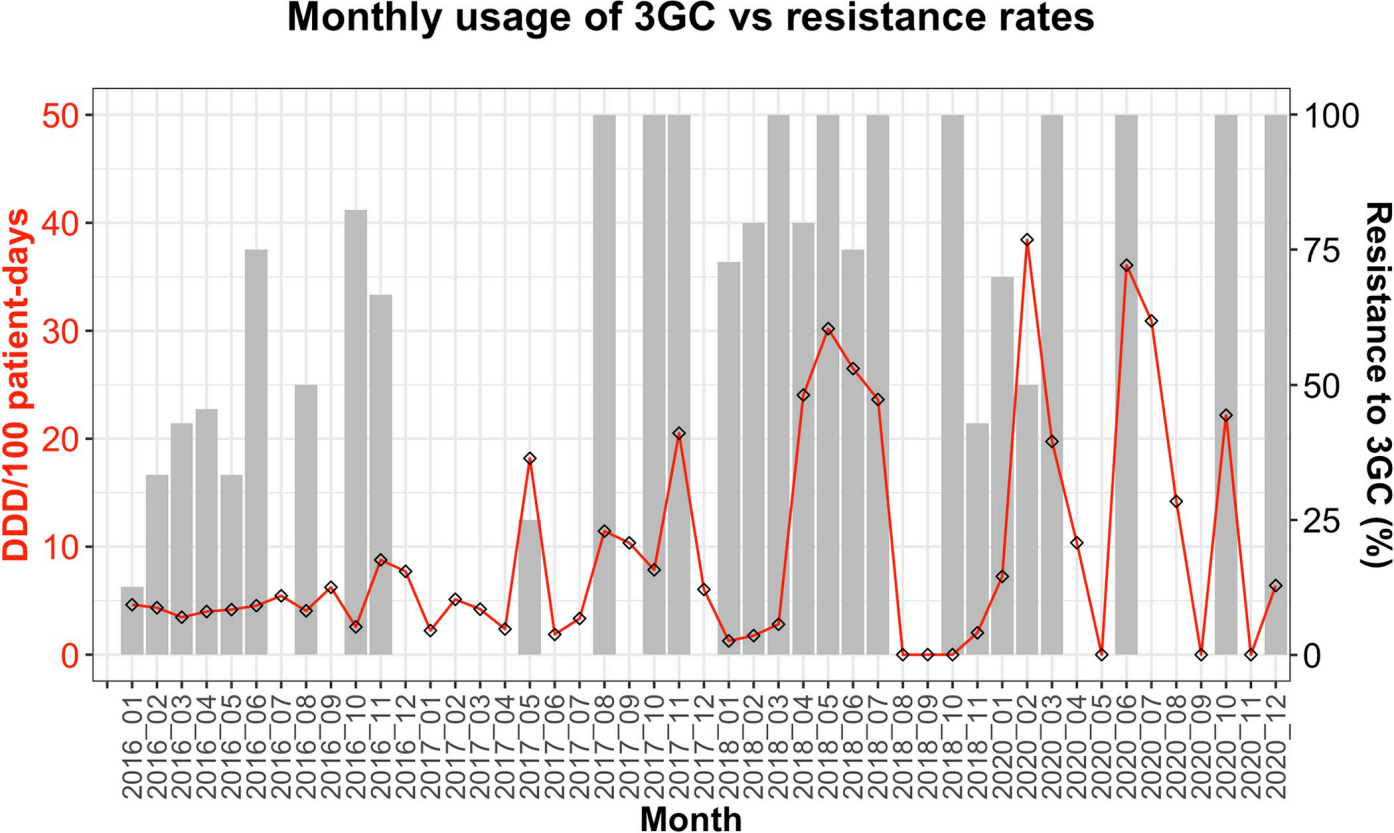
Relationship between 3GC use and resistance. The monthly consumption of 3GCs was expressed as DDD per 100-patient days (red line, left y-axis), while the percentage of *Enterobacterales* resistant to 3GCs (right y-axis) is represented by grey bars.

3GCs are a clinically significant class of antimicrobials because of their effectiveness as first- and second-line drugs in a wide range of bacterial infections. However, the reported high usage of 3GCs at the UTH [27] could drive the emergence of AMR in this vital drug class. To quantify the use of 3GCs, we evaluated the quantities of CTX, CAZ, and CRO dispensed monthly to the medical wards (E-block) for use among inpatients. From the available data (2016, 2017, 2018, and 2020), the most used 3GCs were CTX (226.4 DDDs/100 patient-days) and CRO (223.4 DDDs/100 patient-days), while CAZ (1.5 DDDs/100 patient-days) was rarely used (S7 Table). Notably, there were no records of oral 3GCs being dispensed during the period under review. By visually inspecting the monthly usage and resistance trends in over 15 species belonging to *Enterobacterales* (n = 690), we noted a relationship between 3GC use and resistance. Precisely, increases in 3GC use coincided with high 3GC resistance and vice versa (Fig 7). Furthermore, there was a weak positive correlation between the two variables (r = 0.38, 95% CI = 0.11–0.60).

### Detection of WHO priority pathogens

Under the "critical" priority tier, we found carbapenem-resistant *Acinetobacter baumanii* and *Pseudomonas aeruginosa* at 7.7% (10/130) and 4.4% (20/454), respectively (S8 Table). We also found carbapenem-resistant *Enterobacterales* belonging to various genera, including *Proteus*, *Enterobacter*, *Citrobacter*, and *Salmonella*, with resistance rates ranging from 0.7 to 5.5%. In addition, the species in this priority category showed high levels of 3GC resistance, especially *K*. *pneumoniae* (596/806; 73.9%), *K*. *oxytoca* (55/96; 57.3%), *E*. *coli* (465/847; 54.9%), and *E*. *cloacae* (59/109; 54.1%) (S8 Table).

The "high" priority pathogens included VAN-resistant *Enterococcus faecium* (2/87; 2.3%) and *S*. *aureus* (15/303; 5.0%), MRSA (973/2,181; 44.6%), quinolone-resistant *S*. *enterica* serovar Typhi (43/113; 38.1%), and *Neisseria gonorrheae* resistant to quinolones (7/10; 70%) or 3GCs (2/5; 40%). However, no *Helicobacter* or *Campylobacter* species were reported during the study period. Finally, the "medium" priority tier was represented by PEN-non-susceptible *Streptococcus pneumoniae* (13/66; 19.7%), AMP-resistant *Haemophilus influenzae* (16/45; 35.6%), and two species of fluoroquinolone-resistant *Shigella*, namely *flexneri* (2/93; 5.1%) and *dysenteriae* (3/9; 33.3%).

## Discussion

Using retrospective data (2015–2020) of bacterial isolates from clinical samples at the UTH, we found several Gram-positive and Gram-negative species with high AMR rates that fluctuated over time. Furthermore, MDR was frequent in *E*. *coli*, *K*. *pneumoniae*, and *S*. *aureus*, especially among isolates obtained from blood samples. Importantly, we found a weak positive relationship between monthly 3GC use and resistance among *Enterobacterales* isolated from patients in medical wards.

The high prevalence of MRSA observed in this study coincides with the reported increased use of β-lactams at the UTH [28]. Similarly, studies elsewhere have associated heavy antimicrobial use with MRSA [29]. MRSA produces a *mecA*-encoded penicillin-binding protein (i.e., PBP2a) with a low affinity for β-lactams. However, its susceptibility to various anti-staphylococcal penicillins may vary, making phenotypic characterization challenging. For instance, 3.6% of the cefoxitin-resistant *S*. *aureus* in this study were susceptible to oxacillin (Table 3), consistent with previous findings which suggest that oxacillin is a less reliable predictor of MRSA [30]. Despite the high MDR prevalence among MRSA (Table 3), the MRSA in this study were largely susceptible to non-β-lactam antibiotics. For instance, VAN, the first-line treatment for MRSA infections [31], and LZD, which is associated with better clinical outcomes and fewer adverse effects [32], exhibited high activity against MRSA. However, our results already suggest the emergence of LZD resistance from 2019 (Fig 4).

Among Gram-negative bacteria, *E*. *coli* and *K*. *pneumoniae* exhibited resistance to most antibiotics tested. Notably, 54.9% (465/847) of *E*. *coli* and 73.9% (596/806) *K*. *pneumoniae* isolates were resistant to 3GCs (i.e., CTX) (S8 Table), thus meeting the "critical" category of global pathogens listed by the WHO [19]. In addition, several other *Enterobacterales* exhibited high 3GC resistance, including *K*. *oxytoca* (57.3%), *E*. *cloacae* (54.1%), *E*. *agglomerans* (50.0%), and *Proteus mirabilis* (42.9%). By analyzing monthly ward-level data for 690 *Enterobacterale* isolates, we found that the usage of 3GCs was related with resistance to this drug class. However, our AMU estimates were based on antibiotic doses dispensed by the pharmacy unit, which may not accurately reflect the amounts administered to patients. Therefore, future studies should ascertain AMU by directly reviewing patient drug charts and prescriptions.

The likely mechanism of 3GC resistance could be the production of extended-spectrum β-lactamases (ESBLs) that hydrolyze β-lactam antibiotics, such as CTX. We recently characterized CTX-resistant *Enterobacteriaceae* from the UTH, where we observed the *bla*_CTX-M_ ESBL gene in 97.8% (45/46) isolates [33]. Also, we found other AMR genes encoding resistance to several antibiotics, which could explain the high MDR rates observed in this study. Although MDR in *E*. *coli* and *K*. *pneumoniae* was more common among isolates from blood compared to urine, our dataset lacked information on gender, which could confound the results. For instance, while urinary tract infection (UTI) is generally more common in females [34], UTI complicated by multidrug-resistant ESBL producers is commonly associated with the male gender [35]. Therefore, the proportion of MDR among urine isolates would depend partly on the gender composition of the tested population; thus, stratifying results by gender would give a more accurate comparison. In addition, while the interpretation of manual disk diffusion was standardized and quality-controlled using the automated VITEK 2 compact, the tests depended on disk availability, leading to discrepancies in the number of antibiotics tested (S1 Table). Furthermore, our data lacked bacterial species-disease information as well as details on antibiotic use in non-medical wards. Thus, future studies should be aimed at addressing these shortcomings.

While most *E*. *coli* and *K*. *pneumoniae* isolates in this study were susceptible to carbapenems, IPM resistance increased in 2019. Unfortunately, therapeutic options for carbapenem-resistant *E*. *coli* and *K*. *pneumoniae* were limited, mainly due to the non-availability of alternatives such as colistin. Other "critical" priority pathogens included *A*. *Baumanni* and *P*. *aeruginosa*, resistant to carbapenems, and several *Enterobacteriaceae* species resistant to carbapenems and 3GCs. Furthermore, several "high" and "medium" priority pathogens were detected, including VAN-resistant *E*. *faecium* and fluoroquinolone-resistant *S*. *enterica* serovar Typhi. However, while our study is based on an exhaustive list of all the bacterial isolates processed during the study period, some isolates did not have susceptibility results, limiting our ability to estimate the true prevalence of some priority pathogens. For example, only 31.0% (113/364) of the *S*. *enterica* serovar Typhi isolates were tested for fluoroquinolone resistance. Similarly, only 30.6% (66/216) of the isolated *S*. *pneumoniae* had susceptibility results for AMP. Nevertheless, the frequently isolated pathogens, such as *E*. *coli*, *K*. *pneumoniae*, and *S*. *aureus*, had sufficient AST data to allow valid inferences.

The routine surveillance of AMR may be necessary to design local treatment protocols; however, our results show that such protocols require regular revision for optimal empirical therapy. For instance, clindamycin (CLI) was effective in about 90% of *S*. *aureus* isolates in 2015, but its activity was below 70% in 2020. Despite its nonuse, this reduction in CLI effectiveness could have arisen from cross-resistance with macrolides (e.g., erythromycin), which were dispensed during the period under review (S6 Table). However, confirming this hypothesis requires screening isolates for the *erm* gene, which encodes a target-modifying enzyme to cause resistance to macrolides, lincosamides (e.g., CLI), and streptogramin B [36]. Furthermore, confirmation of other AMR mechanisms will be necessary to implement target-specific AMR control measures effectively.

For example, the observed carbapenem resistance among several Gram-negative species could have been caused by increased active efflux of drugs across the membrane, alterations in the outer membrane porins, or mutations leading to newer PBPs [37]. Equally, resistance could have been due to carbapenemases encoded on plasmids that expand the bacterial host range [38], thus requiring more rigorous control measures than adaptive resistance. The limitations in our data could be addressed by genotypic studies that detect AMR genes and mutations and distinguish clonal AMR spread from horizontal gene transfer. Specifically, there is a need to supplement AST data with WGS to allow robust AMR transmission modeling.

## Conclusion

We analyzed AST data for bacterial species isolated from diverse clinical sources at the UTH between 2015 and 2020. Our results revealed varying AMR rates over the study period, suggesting that antibiograms should be updated regularly based on AMR trends. Furthermore, there was a relationship between 3GC usage and resistance among *Enterobacterales* from patients in medical wards, emphasizing the need to strengthen antimicrobial stewardship. Finally, there were limited treatment options for carbapenem-resistant isolates, highlighting the need to preserve essential antibiotics and explore other last-resort medicines, such as colistin. We anticipate that this study will help guide policy formulation on antimicrobial stewardship.

## Supporting information

S1 Table Clinical sources and AST patterns of bacteria isolated from 2015–2020. (XLSX)

S2 Table Frequency of bacterial species isolated during the study period. (XLS)

S3 Table Distribution of bacterial species among clinical sources. (DOCX)

S4 Table MDR prevalence in various clinical sources. (DOCX)

S5 Table AST pattern of selected bacteria over time. (DOCX)

S6 Table Antimicrobials used at the UTH over the study period. (DOCX)

S7 Table 3GC usage in the medical wards (E-block) in DDDs/100 patient-days. (XLS)

S8 Table AST pattern of WHO priority pathogens. (DOCX)
