# Supplementary material for: Trends, patterns and relationship of antimicrobial use and resistance in bacterial isolates tested between 2015–2020 in a national referral hospital of Zambia
Source: PLoS One. 2024 Apr 16;19(4):e0302053. doi: 10.1371/journal.pone.0302053 (PMC11020921; doi:10.1371/journal.pone.0302053)
Supplement: S3 Table — (DOCX) [file pone.0302053.s003.docx]

**Table S3. Subset of the 28,080 bacterial isolates with source data**

| **Species** | **Number with source data** | **Urine** | **Blood** | **Wound swab** | **Swab; unknown origin** |
| --- | --- | --- | --- | --- | --- |
| *S. aureus* | 3,117 | 118 | 756 | 952 | 903 |
| Coagulase Neg *Staphylococcus* | 2,524 | 202 | 1,197 | 503 | 385 |
| *Enterococcus* species | 1,530 | 862 | 255 | 52 | 67 |
| *E. coli* | 4,545 | 2,684 | 630 | 444 | 463 |
| *K. pneumoniae* | 3,292 | 927 | 1,072 | 370 | 342 |
| Other species | … | … | … | … | … |
| **Total** | **28,080** | **8,082** | **6,728** | **4,328** | **4,222** |

Only selected species and clinical sources are shown here.
