# Supplementary material for: Trends, patterns and relationship of antimicrobial use and resistance in bacterial isolates tested between 2015–2020 in a national referral hospital of Zambia
Source: PLoS One. 2024 Apr 16;19(4):e0302053. doi: 10.1371/journal.pone.0302053 (PMC11020921; doi:10.1371/journal.pone.0302053)
Supplement: S4 Table — (DOCX) [file pone.0302053.s004.docx]

**Table S4. MDR prevalence in various clinical sources**

| **Species** | **Levels** | **MDR**  **“+ve”** | **MDR**  **“-ve”** | **Odds ratio** | **95% CI** | **p-value** |
| --- | --- | --- | --- | --- | --- | --- |
| *E. coli* | Urine | 799 (33.9%) | 1557 | 1 |  |  |
|  | Blood | 294 (52.1%) | 270 | 2.12 | 1.76 – 2.56 | < 0.001^c^ |
| *K. pneumoniae* | Urine | 367 (36.4%) | 642 | 1 |  |  |
|  | Blood | 842 (82.4%) | 180 | 8.18 | 6.66 – 10.05 | < 0.001^c^ |
| *S. aureus* | Wound | 401 (47.1%) | 451 | 1 |  |  |
|  | Blood | 345 (52.7%) | 310 | 1.25 | 1.02 – 1.53 | 0.035^c^ |
|  |  |  |  |  |  |  |

^c^Chi-squared test with Yates' continuity correction
