# Supplementary material for: Trends, patterns and relationship of antimicrobial use and resistance in bacterial isolates tested between 2015–2020 in a national referral hospital of Zambia
Source: PLoS One. 2024 Apr 16;19(4):e0302053. doi: 10.1371/journal.pone.0302053 (PMC11020921; doi:10.1371/journal.pone.0302053)
Supplement: S5 Table — (DOCX) [file pone.0302053.s005.docx]

**Table S5. Antibiotic susceptibility pattern of selected bacteria over time**

| Nitrofurantoin resistance among *E. coli* between 201*7* and 2020 | | | | |
| --- | --- | --- | --- | --- |
| **Year** | **NIT Non-susceptible** | **NIT Susceptible** | **Percent Susceptible (%)** | P-value |
| 2017 | 112 | 435 | 79.5 | Ref |
| 2018 | 129 | 276 | 68.1 | < 0.001^c^ |
| 2019 | 240 | 239 | 49.9 | < 0.001^c^ |
| 2020 | 159 | 101 | 38.8 | < 0.001^c^ |
|  |  |  |  |  |
| Nitrofurantoin resistance among *S. aureus* between 2016 and 2020 | | | | |
| **Year** | **NIT Non-susceptible** | **NIT Susceptible** | **Percent Susceptible (%)** | **P-value** |
| 2016 | 1 | 48 | 98.0 | Ref |
| 2017 | 8 | 146 | 94.8 | 0.690^f^ |
| 2018 | 3 | 34 | 91.9 | 0.310^f^ |
| 2019 | 5 | 42 | 89.4 | 0.108^f^ |
| 2020 | 4 | 22 | 84.6 | 0.046^f^ |
|  |  |  |  |  |
| Cefoxitin resistance among *S. aureus* between 2015 and 2020 | | | | |
| **Year** | **FOX Non-susceptible** | **FOX Susceptible** | **Percent Susceptible (%)** | **P-value** |
| 2015 | 13 | 36 | 73.5 | Ref |
| 2016 | 75 | 181 | 70.7 | 0.826^c^ |
| 2017 | 145 | 272 | 65.2 | 0.321^c^ |
| 2018 | 84 | 108 | 43.8 | 0.042^c^ |
| 2019 | 485 | 344 | 41.5 | < 0.001^c^ |
| 2020 | 194 | 226 | 53.8 | 0.013^c^ |
|  |  |  |  |  |
| Imipenem resistance among *E. coli* between 2018 and 2020 | | | | |
| **Year** | **IPM Non-susceptible** | **IPM Susceptible** | **Percent Susceptible (%)** | **P-value** |
| 2018 | 3 | 112 | 97.4 | Ref |
| 2019 | 11 | 85 | 88.5 | 0.012^f^ |
| 2020 | 5 | 86 | 94.5 | 0.306^f^ |
|  |  |  |  |  |
| Imipenem resistance among *K. pneumoniae* between 2018 and 2020 | | | | |
| **Year** | **IPM Non-susceptible** | **IPM Susceptible** | **Percent Susceptible (%)** | **P-value** |
| 2018 | 5 | 93 | 94.9 | Ref |
| 2019 | 13 | 75 | 85.2 | 0.048^c^ |
| 2020 | 19 | 82 | 81.2 | 0.006^c^ |

^c^Chi-squared test with Yates' continuity correction

^f^Fisher's Exact Test for Count Data
