# Supplementary material for: Trends, patterns and relationship of antimicrobial use and resistance in bacterial isolates tested between 2015–2020 in a national referral hospital of Zambia
Source: PLoS One. 2024 Apr 16;19(4):e0302053. doi: 10.1371/journal.pone.0302053 (PMC11020921; doi:10.1371/journal.pone.0302053)
Supplement: S6 Table — (DOCX) [file pone.0302053.s006.docx]

**Table S6. Antimicrobials used at the UTH over the study period**

| **Drug class** | **Drugs used** |
| --- | --- |
| Penicillins | Penicillin V, Penicillin G, Amoxycillin, Cloxacillin |
| Penicillin combinations | Amoxycillin-Clavulanic acid |
| Narrow-spectrum cephalosporins | Cefalexin, Cefuroxime |
| Third-generation cephalosporins | Cefotaxime, Ceftazidime, Ceftriaxone |
| Carbapenems | Imipenem |
| Aminoglycosides | Gentamicin, Kanamycin |
| Macrolides | Azithromycin, Erythromycin, Clarithromycin |
| Quinolones | Nalidixic acid, Ciprofloxacin, Levofloxacin, Moxifloxacin |
| Amphenicol | Chloramphenicol |
| Tetracycline | Tetracycline, Doxycycline |
| Antifolate | Sulfamethoxazole-Trimethoprim |
| Nitroimidazole | Metronidazole, Tinidazole |
| Oxazolidinones | Linezolid |
| Nitrofuran | Nitrofurantoin |
| Glycopeptide | Vancomycin |
