# Supplementary material for: Trends, patterns and relationship of antimicrobial use and resistance in bacterial isolates tested between 2015–2020 in a national referral hospital of Zambia
Source: PLoS One. 2024 Apr 16;19(4):e0302053. doi: 10.1371/journal.pone.0302053 (PMC11020921; doi:10.1371/journal.pone.0302053)
Supplement: S8 Table — (DOCX) [file pone.0302053.s008.docx]

**Table S8. Antibiotic susceptibility pattern of WHO priority pathogens**

| **Priority** | **Pathogen** | **S** | **I** | **R** | **Total** | **R (%)** |
| --- | --- | --- | --- | --- | --- | --- |
| Critical | *A. baumanii* (carbapenem-resistant) | 117 | 3 | 10 | 130 | 7.7 |
|  | *P. aeruginosa* (carbapenem-resistant) | 419 | 15 | 20 | 454 | 4.4 |
|  | *E. coli* (carbapenem-resistant) | 772 | 22 | 16 | 810 | 2.0 |
|  | *K. pneumoniae* (carbapenem-resistant) | 1299 | 46 | 24 | 1369 | 1.8 |
|  | *P. mirabilis* (carbapenem-resistant) | 173 | 30 | 7 | 210 | 3.3 |
|  | *E. agglomerans* (carbapenem-resistant) | 152 | 4 | 4 | 160 | 2.5 |
|  | *E. cloacae* (carbapenem-resistant) | 137 | 10 | 1 | 148 | 0.7 |
|  | *K. oxytoca* (carbapenem-resistant) | 76 | 3 | 1 | 80 | 1.3 |
|  | *C. diversus* (carbapenem-resistant) | 60 | 5 | 2 | 67 | 3.0 |
|  | *C. freundii* (carbapenem-resistant) | 54 | 5 | 3 | 62 | 4.8 |
|  | *S.* Typhi (carbapenem-resistant) | 51 | 1 | 3 | 55 | 5.5 |
|  | *E. coli* (3GC-resistant) | 344 | 38 | 465 | 847 | 54.9 |
|  | *K. pneumoniae* (3GC-resistant) | 179 | 31 | 596 | 806 | 73.9 |
|  | *P. mirabilis* (3GC-resistant) | 146 | 15 | 121 | 282 | 42.9 |
|  | *E. agglomerans* (3GC-resistant) | 105 | 13 | 118 | 236 | 50.0 |
|  | *E. cloacae* (3GC-resistant) | 45 | 5 | 59 | 109 | 54.1 |
|  | *K. oxytoca* (3GC-resistant) | 39 | 2 | 55 | 96 | 57.3 |
|  | *C. diversus* (3GC-resistant) | 33 | 11 | 24 | 68 | 35.3 |
|  | *C. freundii* (3GC-resistant) | 32 | 5 | 26 | 63 | 41.3 |
|  | *S.* Typhi (3GC-resistant) | 51 | 4 | 7 | 62 | 11.3 |
| High | *E. faecium* (vancomycin-resistant) | 85 | 0 | 2 | 87 | 2.3 |
|  | *S. aureus* (vancomycin-resistant) | 284 | 4 | 15 | 303 | 5.0 |
|  | *S. aureus* (methicillin-resistant) * | 1178 | 30 | 973 | 2181 | 44.6 |
|  | *S.* Typhi (fluoroquinolone-resistant) | 74 | 27 | 43 | 113 | 38.1 |
|  | *N. gonorrhoeae* (fluoroquinolone-resistant) | 2 | 1 | 7 | 10 | 70.0 |
|  | *N. gonorrhoeae* (3GC-resistant) | 3 | 0 | 2 | 5 | 40.0 |
| Medium | *S. pneumoniae* (penicillin-non-susceptible) | 53 | 1 | 12 | 66 | 19.7* |
|  | *H. influenzae* (ampicillin-resistant) | 27 | 2 | 16 | 45 | 35.6 |
|  | *Shigella flexneri* (fluoroquinolone-resistant) | 36 | 1 | 2 | 39 | 5.1 |
|  | *Shigella dysenteriae* (fluoroquinolone-resistant) | 6 | 2 | 3 | 9 | 33.3 |
